# Dr. Edward Whinery

**Published:** 1869-02

**Authors:** 


### OBITUARY. 1869

Since the last issue of the Journal our profession has been deprived of one of its brightest ornaments. Death has taken from our midst Dr. Edward Whinery, of Ft. Madison, in this county, one of the oldest practitioners in the State. A man of high professional attainments and untiring in his energy for the promotion of Medical Science. May his memory live in the hearts of his many friends.

